# StreptInCor: A Candidate Vaccine Epitope against *S. pyogenes* Infections Induces Protection in Outbred Mice

**DOI:** 10.1371/journal.pone.0060969

**Published:** 2013-04-08

**Authors:** Edilberto Postol, Raquel Alencar, Fabio T. Higa, Samar Freschi de Barros, Lea M. F. Demarchi, Jorge Kalil, Luiza Guilherme

**Affiliations:** 1 Heart Institute, School of Medicine, University of São Paulo, São Paulo, Brazil; 2 Institute for Immunology Investigation, National Institute of Science and Technology, University of São Paulo, São Paulo, Brazil; 3 Clinical Immunology and Allergy Division, School of Medicine, University of São Paulo, São Paulo, Brazil; University of South Dakota, United States of America

## Abstract

Infection with *Streptococcus pyogenes* (*S. pyogenes*) can result in several diseases, particularly in children. *S. pyogenes* M protein is the major virulence factor, and certain regions of its N-terminus can trigger autoimmune sequelae such as rheumatic fever in susceptible individuals with untreated group A streptococcal pharyngitis. In a previous study, we utilized a large panel of human peripheral blood cells to define the C-terminal protective epitope StreptInCor (medical identity), which does not induce autoimmune reactions. We recently confirmed the results in HLA-transgenic mice. In the present study, we extended the experimental assays to outbred animals (Swiss mice). Herein, we demonstrate high titers of StreptInCor-specific antibodies, as well as appropriate T-cell immune responses. No cross-reaction to cardiac myosin was detected. Additionally, immunized Swiss mice exhibited 87% survival one month after challenge with *S. pyogenes*. In conclusion, the data presented herein reinforce previous results in humans and animals and further emphasize that StreptInCor could be an effective and safe vaccine for the prevention of *S. pyogenes* infections.

## Introduction

Group A streptococci (GAS) cause several human diseases, including common infections, such as pharyngitis, scarlet fever and impetigo, and more complex and invasive diseases, such as necrotizing fasciitis and streptococcal toxic shock syndrome. Moreover, GAS infections can lead to the non-suppurative sequelae of glomerulonephritis and rheumatic fever (RF). Rheumatic heart disease (RHD), which is the most important manifestation of RF, may lead to cardiac valve lesions. The broad range of GAS pathologies is related to the adaptation of GAS to the diverse physiologic conditions presented by the human host as well as to the different infection sites [1].

An estimated 616 million new cases of pharyngitis occur each year, with more than 500,000 deaths occurring due to serious GAS diseases around the world. The worldwide estimated incidence of RHD is at least 15.6 million cases/year [2]. In Brazil, it is estimated that 10 million streptococcal pharyngitis cases occur each year, of which approximately 30,000 cases will develop RF and 15,000 could progress to RHD [3].

Penicillin-benzathine is routinely used to treat GAS infections and RF [4]; however, in some cases, it fails to effectively treat GAS [5]. Numerous studies have aimed at designing a vaccine using different streptococcal antigens as the immunogenic targets [6]. To prevent RF, RHD and other invasive diseases caused by *S. pyogenes* infections, several M protein-based vaccines have been studied. The GAS M protein is composed of two polypeptide chains in an α-helical coiled-coil shape anchored within the cellular membrane of the bacteria. The N-terminal region of the M protein (A repeat) is highly polymorphic and antigenic and defines the GAS serotypes, of which approximately 200 have been described to date. The C-terminal portion of the M protein is conserved among the different GAS serotypes [7]. Previously, a multivalent vaccine was constructed by combining sequences from the N-terminal portions of the 6 GAS strains found most frequently in the US into a recombinant protein designed to evoke a specific type of immune response. This vaccine was well tolerated by healthy volunteers when administered in a phase I human clinical trial [8]. A similar approach was employed to produce and test a vaccine containing sequences from 26 M proteins most frequently found in Europe and North America [9], and recently, more promising results were obtained with a 30-valent M Protein based vaccine [10]. Furthermore, the conserved C-terminal region of the streptococcal M protein has also been studied in the development of vaccines capable of conferring protection against the majority of GAS strains [11]–[16].

Using 20 years’ worth of knowledge about the development of autoimmune reactions [17]–[19], we searched for C-terminus-derived protective peptides with the ability to induce wide protection against GAS strains without causing autoimmune reactions and disease. Briefly, we evaluated the humoral and cellular reactivity of 79 overlapping synthetic peptides (20-mers) that are derived from the C-terminal region of the M5 protein and that differ by a single amino acid residue. Human sera from 620 individuals and 260 PBMC samples allowed us to define the immunodominant T and B cell epitopes, which were composed of 22 and 25 amino acid residues, respectively. A 55-amino-acid candidate peptide named StreptInCor (medical identity), which included both the T and B cell epitopes linked by 8 amino acid residues (similar to the natural sequence of M5 protein), was constructed [20]–[21]. Recently, structural stability studies have shown that the StreptInCor peptide is highly stable, is recognized by the T cell receptor in the context of any HLA class II molecule and leads to consequent activation of T helper cells [22]. StreptInCor was capable of inducing a strong immune response in HLA class II transgenic mice without causing autoimmune or deleterious reactions up to one year after the immunizations [23]. After promising results observed by using mice with the same genetic background (isogenic BALB/c mice) [21] and specific human HLA class II (transgenic mice) [23], we aimed to verify if this vaccine candidate model could induce a good immune response between animals with diverse genetic background such as outbred Swiss mice, since these animals present genetic polymorphisms that could reflect the human population more efficiently.

## Materials and Methods

### 1. StreptInCor

The synthetic StreptInCor peptide (KGLRRDLDASREAKKQLEAEQQKLEEQNKISE-ASRKGLRRDLDASREAKKQVEKA) was synthesized using a 9-α-fluorenylmethoxy-carbonyl (Fmoc) solid-phase strategy and purified by reverse phase high-pressure liquid chromatography (RP-HPLC, Shimadzu, Japan) as previously described [19]. Peptide quality was assessed by matrix-assisted desorption ionization mass spectrometry (MALDI-ToF, Ettan Maldi Tof Pro, Amersham-Pharmacia, Uppsala, Sweden).

### 2. Animals

Specific pathogen-free 6- to 8-week-old female Swiss mice were obtained from CEMIB (Unicamp, Campinas, Brazil) and maintained and handled in the animal facility at the Tropical Medicine Institute, University of São Paulo, Brazil. The mice were housed in autoclaved cages (Alesco, Brazil) and handled under sterile conditions. All procedures were performed in accordance with the Brazilian Committee for animal care and use (COBEA) guidelines and approved by the Tropical Medicine Institute Ethics Committee for animal research (project number 002/08).

### 3. Immunization

Groups of 6 mice were subcutaneously immunized twice, 14 days apart, with an emulsion containing 10 µg of StreptInCor adsorbed onto 60 µg of aluminum hydroxide gel (Sigma-Aldrich Corp., St. Louis, MO, USA) in saline. The control animals received 60 µg of aluminum hydroxide gel in saline. Mouse sera were obtained 14 days after the second boost by retro-orbital puncture following light anesthesia.

### 4. Serum Antibody Measurements

Serum antibody titers were quantified using ELISA. Briefly, 1 µg of StreptInCor, M1 recombinant protein produced and purified in our laboratory (clone kindly provided by Prof. Patrick Cleary, University of Minnesota Medical School, MN, USA), and porcine cardiac and muscle myosin (Sigma, USA) were diluted in coating buffer (0.05 M carbonate-bicarbonate, pH 9.6, 50 µL/well) and plated onto a 96-well MaxiSorp assay plate (Nunc, Denmark). After overnight incubation, the plates were blocked with 0.25% gelatin (Sigma) and 0.05% Tween-20 (Sigma) in PBS (dilution buffer) for 1 h at room temperature. Serial 2-fold dilutions of the sera in dilution buffer, starting at 1∶100, were added to the plates (50 µL/well). After a 2-h incubation at 37°C and three washes (200 µL/well) with 0.05% Tween-20 in PBS (rinse buffer), the plates were incubated with peroxidase-conjugated anti-mouse IgG (Pharmingen, San Diego, CA, USA) diluted 1∶2000 in dilution buffer (50 µL/well) for an hour at 37°C. To measure the anti-StreptInCor IgG isotypes, different plates (to each isotype) were sequentially treated with biotinylated anti-mouse IgG1, IgG2a, IgG2b and IgG3 (Pharmingen) diluted to 2 µg/mL (50 µL/well, 1 h at 37°C) and then were washed three times (200 µL/w) with rinse buffer and incubated with streptavidin peroxidase (Pharmingen) at a 1∶1000 dilution (50 µL/well, 1 h at 37°C). Next, the plates were washed three times (200 µL/w) with rinse buffer, and the reaction was carried out with 50 µL/w of 0.4 mg/ml orthophenylenediamine (OPD, Sigma) in 100 mM sodium citrate (Merck, Germany) containing 0.03% H_2_O_2_ (Merck). After 10 minutes at room temperature, the reactions were stopped with 4 N H_2_SO_4_, and the optical density was evaluated using a 490 nm ELISA filter in an MR4000 ELISA plate reader (Dynatech, Chantilly, VA, USA). The plates were washed three times with rinse buffer (200 µL/w) between incubations. Endpoint titers were defined as the reciprocal of the highest dilution giving an absorbance higher than two standard deviations above the mean background obtained with non-immune mice sera diluted at 1∶100.

### 5. Proliferation Assays

For proliferation assays, 2.5×10^5^ splenocytes were cultured in 96-well Nunclon plates (Nunc, Denmark) with StreptInCor peptide (1 and 10 µg/ml), cardiac myosin (10 µg/ml), muscle myosin (10 µg/mL) and Concanavalin A (5 µg/ml, Sigma, USA) in RPMI-1640 (Gibco, Grand Island, NY, USA) supplemented with 2 mM L-glutamine (Sigma), 10 mM Hepes (Sigma), 5% fetal calf serum (Gibco), 40 µg/mL gentamicin and 20 µg/mL peflacin for 96 h at 37°C in a humidified 5% CO_2_ incubator (Forma Scientific, USA). All of the antigens were tested in triplicate, and the cells were pulse-labeled with 0.5 µCi 3H-thymidine/well (Amersham-Pharmacia, England, UK) for the final 18 h of culture. The cells were then harvested, and the proliferation was measured by thymidine incorporation using an automated beta counter (Betaplate, Wallac-PerkinElmer, USA). The proliferative response was considered positive when the stimulation index (SI) was ≥ 2.0. The stimulation index (SI) was defined as the experimental counts per minute (cpm) divided by the negative control (non-stimulated cells) counts per minute.

### 6. M Type 1 *S. pyogenes*

The M type 1 isolate was obtained from a patient with an oropharyngeal infection who was treated at the Clinical Hospital of the Medical School, University of São Paulo, Brazil. This sample was obtained from the collection of *S. pyogenes* strains belonging to the Microbiology Laboratory of the Clinical Hospital and this use was approved by the National Research Ethics (CONEP n° 0646/47). The identification of *S. pyogenes* was based on characteristic hemolysis in blood agar and sensitivity to bacitracin. M type 1 *S. pyogenes* was identified by *emm* gene amplification and sequencing, followed by analysis in the BLAST2 databank (National Center for Biotechnology Information, available at http//www.ncbi.nlm.nih.gov/BLAST; and CDC, Department of Health and Human Services, Centers for Disease Control and Prevention, at http//www.cdc.gov/ncidod/biotech/strep).

### 7. Streptococcal Adhesion and Invasion Assay

Semi confluent monolayers of HEp2 cells (human larynx carcinoma, ATCC CCL23) were cultured in Dulbecco’s Modified Eagle Medium (D-MEM, Gibco BRL, Paisley, UK) that was supplemented with 1 mM L-glutamine (Sigma) and 10% fetal calf serum (Gibco). After being washed three times with PBS, the monolayers were infected with approximately 2.5×10^9^ CFU of M type 1 *S. pyogenes* that was previously incubated with StreptInCor immunized mouse sera (1∶2000) or sera from the animals injected with PBS (control, 1∶2000 dilution) for 1 h at 37°C. After 120 min at 37°C and 5% CO_2_, the cells were washed once with PBS, detached from the wells by the addition of 200 µl of trypsin and lysed with 800 µl of cold sterile distilled water. The lysates were diluted appropriately and plated on blood agar. Adhesion inhibition percentage was calculated based on the difference between the CFUs from the cultures treated with the sera from immunized and control mice.

### 8. Challenge

Swiss mice immunized with StreptInCor and controls were challenged with M type 1 *S. pyogenes* by intraperitoneal injection of 1.5×10^7^ CFU/100 µL (lethal dose, 50%). The animals were housed five per cage with members of the same group. The mice were monitored daily, and the mortality of the vaccinated and control mice was recorded. The results were presented as the percentage of mice surviving for 30 days. These experiments were performed in accordance with the Brazilian Committee for animal care and use (COBEA) guidelines and approved by the Tropical Medicine Institute Ethics Committee for animal research. All surviving mice appeared healthy at 30 days after challenge.

### 9. Histopathologic Analysis

Four micro-thick tissue sections from the heart, kidneys, liver, spleen, joints and brain of the immunized and control mice were collected, immediately placed in PBS containing 10% formaldehyde, paraffin-processed and stained with hematoxylin and eosin as previously described [23].

### 10. Statistical Analysis

Mann-Whitney and Dunn’s tests were used to compare the differences observed between StreptInCor-immunized and control groups. Challenge assays were analyzed using the log-rank (MantelCox) test. The data were analyzed using Graph Pad Prism version 5.01 for Windows (Graph Pad Software, San Diego California USA, www.graphpad.com). *P* values ≤0.05 were considered significant.

## Results

### 1. StreptInCor is Able to Induce High Specific IgG Antibody Titers with neither Cross-reaction with Cardiac Myosin nor Deleterious Reactions

**Figure 1:**
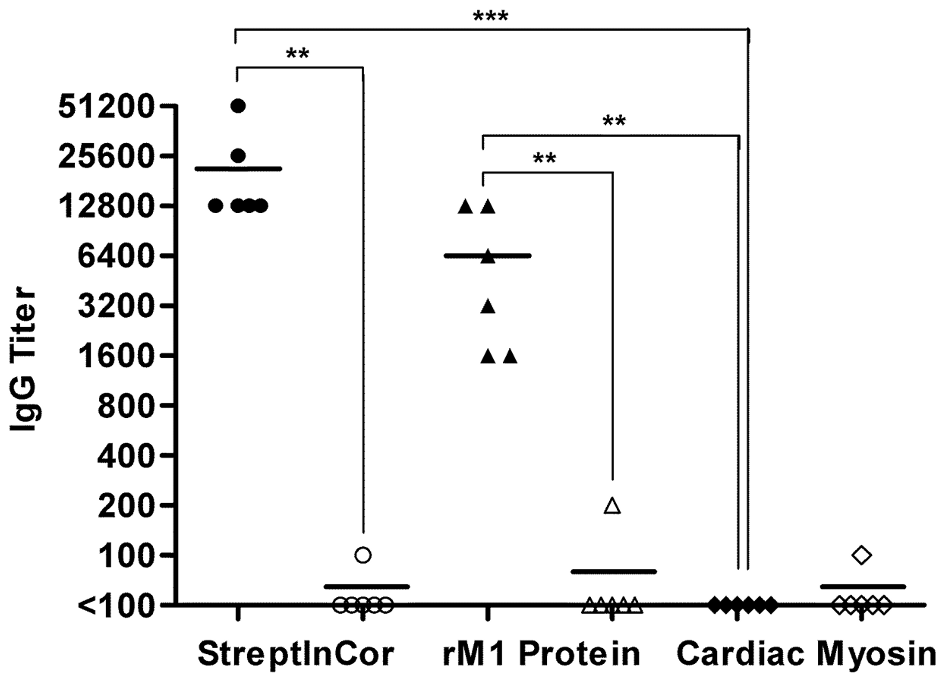
StreptInCor did not induce cross-reactive antibodies against cardiac myosin. Each data point represents the titer for each sample, and the bar represents the mean. Specific anti-StreptInCor IgG antibodies from immunized mice (black circles) also recognized rM1 protein (black triangles) but did not react with cardiac myosin (black diamonds). Control animals received only aluminum hydroxide and did not react against StreptInCor (open circles), rM1 protein (open triangles) or cardiac myosin (open diamonds). Endpoint titers were defined as the reciprocal of the highest dilution giving an absorbance higher than two standard deviations above the mean background obtained with non-immune mice sera diluted at 1∶100. *P* values: *** (≤0.001); ** (≤0.01) and * (≤0.05).

The production of high titers of StreptInCor-specific antibodies was observed among immunized Swiss mice (1∶12,800 to 1∶51,200), whereas no reactivity was observed among control mice (*P*≤0.01) (Figure 1). The sera of immunized mice were also able to recognize the heterologous rM1 protein with titers ranging from 1∶1,600 to >1∶12,800 (*P*≤0.01). No reactivity against cardiac myosin was detected (Figure 1) (*P*≤0.001). In addition, the histopathologic analysis of these mice 30 days after immunization demonstrated no autoimmune or deleterious reactions in the heart, kidneys, liver, spleen, joints or brain (data not shown).

**Figure 2:**
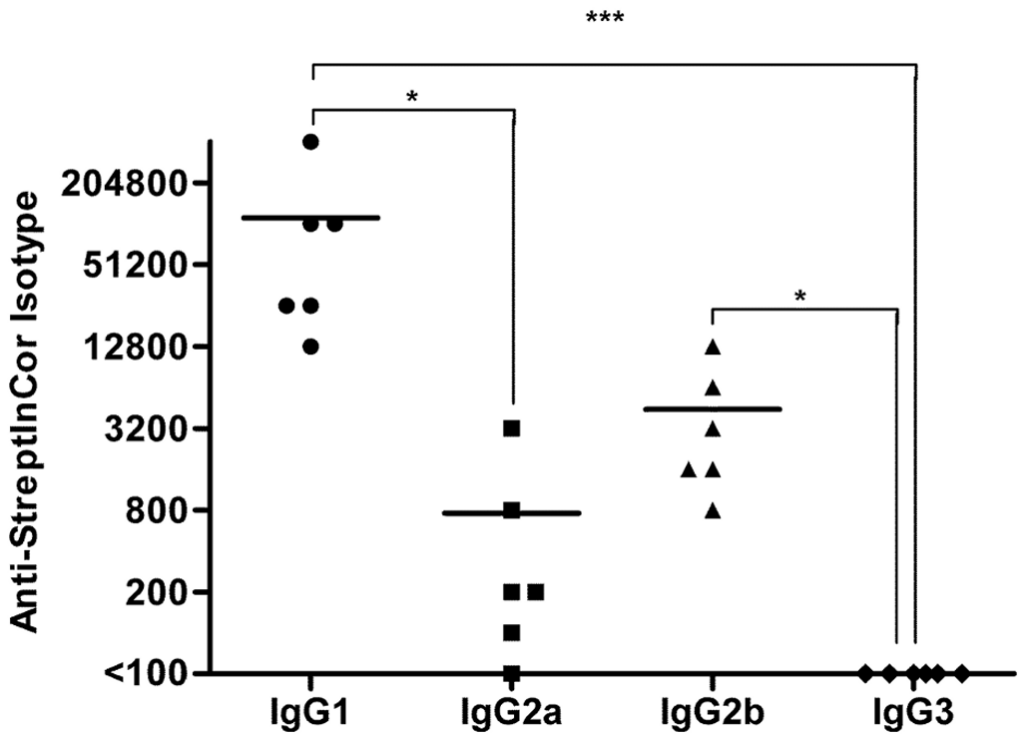
Anti-StreptInCor specific IgG are mainly of IgG1 isotype. Sera from Swiss mice (n = 6) immunized with 10 µg of StreptInCor adsorbed onto 60 µg of aluminum hydroxide were tested to evaluate the production of IgG isotypes against StreptInCor: IgG1 (black circles); IgG2a (black squares); IgG2b (black triangles); IgG3 (black diamonds). Each data point represents the titer for each sample, and the bar represents the mean. Negative samples: (titers <100). Endpoint titers were defined as the reciprocal of the highest dilution giving an absorbance higher than two standard deviations above the mean background obtained with non-immune mice sera diluted at 1∶100. *P* values: *** (≤0.001); * (≤0.05).

Furthermore, we evaluated the IgG isotypes specific for StreptInCor. Sera from immunized mice demonstrated a predominance of the anti-StreptInCor IgG1 isotype compared to the IgG2a (*P*≤0.05) and IgG3 isotypes (*P*≤0.001), as well as a higher reactivity of IgG2b compared to IgG3 (*P*≤0.05) (Figure 2).

### 2. StreptInCor Induces Splenocyte Proliferation *in vitro*

**Figure 3:**
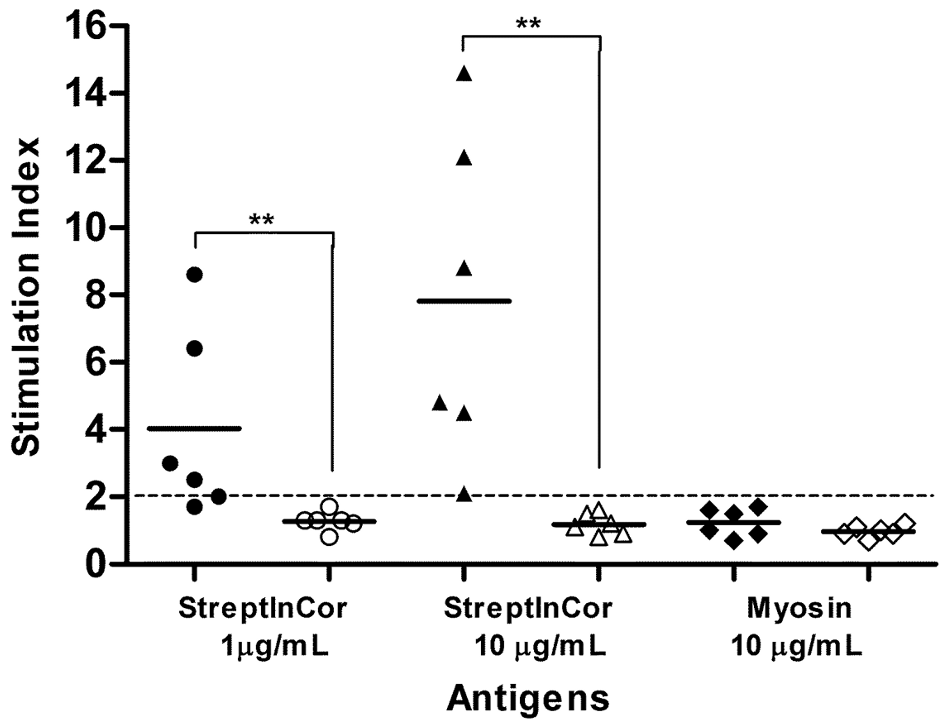
StreptInCor did not induce T-cell cross reactivity against cardiac myosin. Splenocytes from Swiss mice (n = 6) immunized with 10 µg of StreptInCor adsorbed onto 60 µg of aluminum hydroxide (black symbols) or injected with aluminum hydroxide alone (controls, open symbols) were incubated with StreptInCor at 1 µg/mL (circles), 10 µg/mL (triangles) or cardiac myosin (diamonds). Stimulation indices ≥2.0 (dotted line) were considered positive. Each data point represents a sample, and the line represents the mean. *P* values: ** (≤0.01).

Splenocytes from immunized Swiss mice exhibited an *in vitro* dose–dependent cellular immune response to StreptInCor. We observed a positive immune response in 5 out of 6 mice (SI ≥2.0) during the treatment of splenocytes with 1 µg/mL of StreptInCor and an increase of proliferative response after treatment with 10 µg/mL of StreptInCor (Figure 3). No cellular immune response against cardiac myosin was observed (*P*≤0.01) (Figure 3). The cells from control mice did not proliferate in response to specific antigens; however, strong proliferation in response to Concanavalin A (SI ≥15.0) (data not shown) was observed for both immunized and control mice.

### 3. The Sera from Immunized Mice Inhibit Bacterial Adhesion and Invasion into HEp-2 Cells

We evaluated the ability of sera from StreptInCor immunized mice and controls to inhibit M1 bacterial adhesion of pharyngeal epithelial cells (HEp-2). Our results showed that the polyclonal sera from Swiss mice immunized with StreptInCor inhibited adhesion and invasion of M1 strain (63.08% ±1.01) when compared with control groups (3.88% ±4.21) (*P*<0.05).

### 4. StreptInCor Promotes Protection against the S. pyogenes M1 Strain

**Figure 4:**
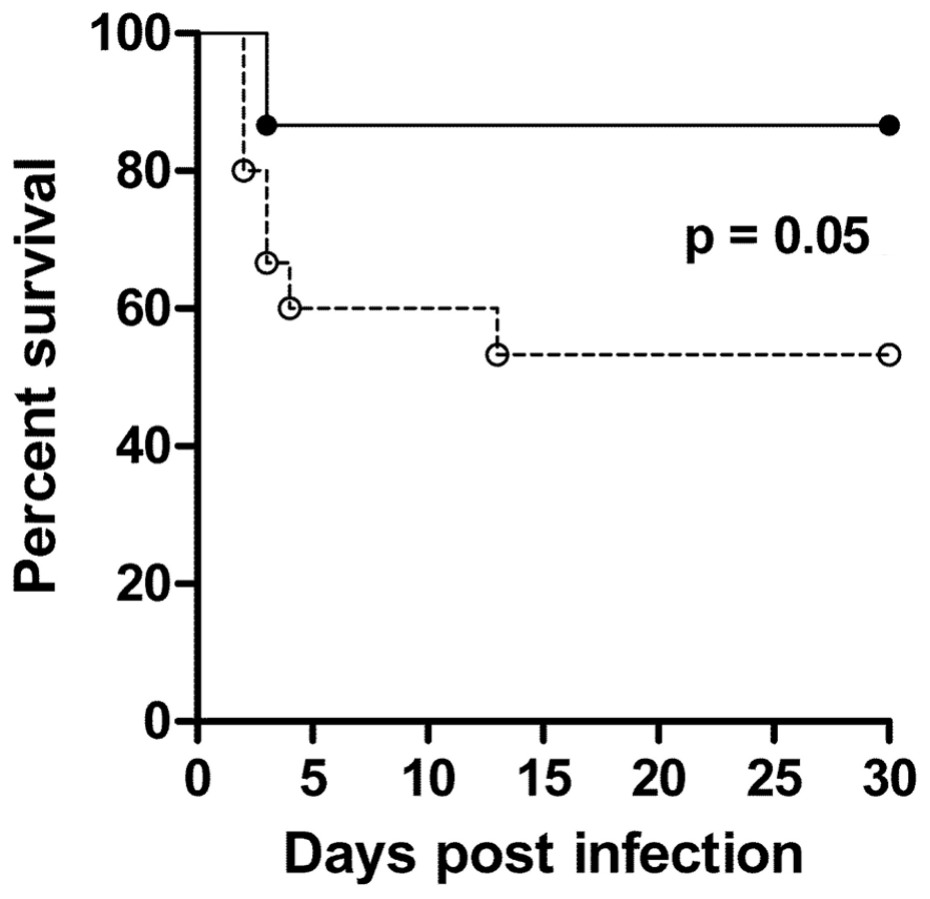
StreptInCor promotes long-lasting survival. Swiss mice (n = 15) from three independent experiments immunized with 10 µg of StreptInCor adsorbed onto 60 µg of aluminum hydroxide (black circles) were protected against *S. pyogenes* infection in comparison to controls (n = 15) that received only the adjuvant (open circles). The log rank test showed that differences between StreptInCor-immunized Swiss and control groups were statistically significant (*P*<0.05).

The vaccinated Swiss mice were challenged with a virulent M1 strain of *S. pyogenes*. Figure 4 shows the results of three different experiments. Among the StreptInCor-immunized Swiss mice, 87% survived for 30 days, whereas 53% of non-vaccinated mice died during the first 13 days following the challenge (*P*≤0.05) (Figure 4).

## Discussion

The development of an effective and safe *S. pyogenes* vaccine is an important issue, and several research groups are working towards this goal. GAS-related diseases remain an important public health problem, especially in developing countries [3], [24]–[26]. A vaccine that is capable of inducing protection against *S. pyogenes* will be not only important for the treatment of pharyngitis but also potentially effective in the treatment of several other more complex diseases [27].

The results presented herein demonstrate a strong protective immune response in outbred mice (Swiss). This conclusion is based on (1) the strong and specific cellular immune response, (2) the high titers of specific antibodies induced by immunization, (3) the ability of StreptInCor specific antibodies to inhibit the adhesion of the *emm1 S. pyogenes* strain *in vitro,* and (4) the four-week survival of immunized Swiss mice injected with the virulent *emm1* strain.

As expected from the use of the aluminum hydroxide gel as adjuvant, the immunized mice showed a dominant IgG1 production (IL-4-dependent) as opposed to IgG2a (IFN-γ-dependent). However, the titers of IgG2a and IgG2b anti-StreptInCor were higher than that observed between HLA class II transgenic mice, suggesting a more balanced production of T-dependent antibodies than the observed in the transgenic models, where only human MHC is presenting antigens to mouse T lymphocytes [23], [28]. Aluminum compounds have been used in several licensed human vaccines for several decades and have been reported to be safe [29]. However, the effects of aluminum salts on B-cell priming *in vivo* (resulting in antibody production) have been attributed to IL-4 secreted by a myeloid cell population and do not involve activated Th2 lymphocytes [30]. In spite of this, StreptInCor vaccination induced a very good specific immune response in Swiss mice. As seen with antibodies, the splenocytes from vaccinated Swiss mice showed a strong and specific proliferative response to StreptInCor, without reacting against cardiac myosin. These results, together with our previous studies [23], imply long lasting immune response mediated by both T cells and antibodies. Moreover, the antibodies raised against StreptInCor were able to recognize a heterologous M protein (rM1). The ability of specific antibodies to identify the StreptInCor sequence within the whole heterologous rM1 protein and inhibit *S. pyogenes* adhesion and invasion to HEp-2 cells suggests that anti-StreptInCor antibodies are capable of recognizing the StreptInCor epitope in the context of the whole protein and also to inhibit *S. pyogenes* adhesion to HEp-2 cells, indicating an important role of antibodies against bacterial colonization.

In susceptible individuals, *S. pyogenes* infections can lead to autoimmune damage, and the understanding of the mechanisms of autoimmunity has advanced greatly in the past 20 years [17], [31]. Therefore, we gave special consideration to the safety of StreptInCor immunization. Cardiac myosin is the most abundant protein in the heart tissue. The recognition of several cardiac myosin epitopes by both T cells and antibodies is thought to be involved in the pathogenesis of rheumatic carditis [32]–[35]. We did not observe neither cellular nor humoral reaction against cardiac myosin, indicating absence of cross reactivity against cardiac tissue. Also, we did not detect any deleterious reactions against several organs (data not shown). These results are in agreement with those recently described for HLA-class II transgenic mice, in which no autoimmune reactions or heart-tissue protein cross-reactive antibodies were detected in several organs [23].

It is of interest to note that Swiss mice responded well to immunization with lower StreptInCor doses (1 µg and 10 µg), whereas the HLA class II transgenic mice required higher doses (50 µg) [23]. This difference may be explained by the presence of only one specific MHC molecule on the surface of the antigen-presenting cells (APCs) of HLA class II transgenic mice instead of the two naturally expressed MHC molecules (I-A and I-E) present in Swiss mice.

Furthermore, StreptInCor and the C-terminal region of the *emm1* type are 72% homologous, which ensured that the peptides processed from StrepInCor in antigen-presenting cells were able to induce a protective immune response. These results are in agreement with previous data described for both humans and transgenic mice [22]–[23].

The development of an effective and safe *S. pyogenes* vaccine is particularly challenging because there are more than 200 strains of *S. pyogenes*. In addition, the strains linked to mortality and morbidity vary regionally, as in the cases of Belgium and Brazil [36], indicating the need for a vaccine with broad coverage [37].

Thus far, the more advanced studies on vaccine development for *S. pyogenes* have focused on a multivalent protein based on the 26 most frequent GAS serotypes in the USA [8]–[9]. However, this construct does not cover all of the serotypes involved in morbidity and mortality [38]. To circumvent these difficulties, an N-terminus-based vaccine was first developed using the 26 most frequent GAS strains in the USA. This was followed by another 30-valent vaccine construction that was immunogenic in rabbits, inducing significant levels of bactericidal antibodies against the specific vaccine serotypes plus 24 different GAS strains.

In addition, vaccines based on the highly conserved M protein C-terminal region have also been designed [14]–[16]. Our vaccine model (StreptInCor) incorporates the 55-amino-acid sequence from the M5 protein C-terminal region and encompasses epitopes for both human B and T cells [21].

In conclusion, the data presented herein indicate that StreptInCor is able to induce a robust protective immune response in outbred mice. Studies aimed at further evaluation of this vaccine candidate are under way, with the goals of obtaining a good mucosal immune response in different species and verifying the coverage of different *S. pyogenes* strains.
